# Bulliform Phytolith Research in Wild and Domesticated Rice Paddy Soil in South China

**DOI:** 10.1371/journal.pone.0141255

**Published:** 2015-10-21

**Authors:** Xiujia Huan, Houyuan Lu, Can Wang, Xiangan Tang, Xinxin Zuo, Yong Ge, Keyang He

**Affiliations:** 1 Institute of Geology and Geophysics, Chinese Academy of Sciences, Beijing, China; 2 University of Chinese Academy of Sciences, Beijing, China; 3 Center for Excellence in Tibetan Plateau Earth Science, Chinese Academy of Sciences, Beijing, China; 4 Soil & Fertilizer and Environmental & Resources Research Institute, Jiangxi Academy of Agricultural Sciences, Nanchang, Jiangxi Province, China; Chinese Academy of Sciences, CHINA

## Abstract

Bulliform phytoliths play an important role in researching rice origins as they can be used to distinguish between wild and domesticated rice. Rice bulliform phytoliths are characterized by numerous small shallow fish-scale decorations on the lateral side. Previous studies have shown that domesticated rice has a larger number of these decorations than wild rice and that the number of decorations ≥9 is a useful feature for identifying domesticated rice. However, this standard was established based on limited samples of modern rice plants. In this study, we analyzed soil samples from both wild and domesticated rice paddies. Results showed that, in wild rice soil samples, the proportion of bulliform phytoliths with ≥9 decorations was 17.46% ± 8.29%, while in domesticated rice soil samples, the corresponding proportion was 63.70% ± 9.22%. This suggests that the proportion of phytoliths with ≥9 decorations can be adopted as a criterion for discriminating between wild and domesticated rice in prehistoric soil. This indicator will be of significance in improving the application of fish-scale decorations to research into rice origins and the rice domestication process.

## Introduction

Rice (*Oryza sativa*) is among the world’s most important and ancient domesticated crops [1]. There is still controversy regarding when rice cultivation and domestication started [2, 3]. Some researchers believe that rice was domesticated 9,000–10,000 years ago, based on evidence from archaeological rice fossils and rice DNA [4–10], while others argue that the process of rice domestication is only known to have begun with certainty 8,000–7,700 years ago, based on the study of unearthed rice spikelet bases [11, 12]. One cause of this disagreement is the lack of unified standards for distinguishing between wild and domesticated archaeological rice remains, with this remaining an urgent problem in the research of rice origins [13].

Traditionally, charred rice grains and spikelet bases from archaeological sites have been employed to identify wild/domesticated rice remains [4, 11, 12, 14]. Past research on rice grain morphology has shown that a wild rice grain is thinner and longer than a domesticated grain, which indicates that it has a greater length-width ratio (with a ratio boundary of 3.5) [15]. The spikelet bases of domesticated rice can be identified by their uneven profile, dimpled appearance, and less symmetrical scars. In contrast, wild-type rice spikelet bases typically have a straight profile at their base, and shattering results in a smooth and round abscission scar, with a small, distinct vascular pore [11, 16]. However, the application of these indictors to archaeological remains still leaves a degree of uncertainty [2]. Liu et al. [2] have proved that the size and shape of rice grains are not reliable identifiers for distinguishing domesticated grains from wild grains; in addition, charred remains and spikelet bases are heavily dependent on unique burial conditions and preservation processes.

To date, phytolith analysis has been a crucial method for identifying rice remains uncovered from archaeological sites and sediments. Rice plants produce three distinctive phytoliths: bilobate phytoliths from rice leaves and stems, double-peaked phytoliths from the rice husk, and bulliform phytoliths from the rice leaves [17]. The bilobate phytolith is typical of the Oryzoideae subfamily and contrasts with the characteristic features of *Oryza* plants [18]. However, measurement of bilobates does not enable discrimination of cultivated and wild *Oryza* species [19]. Double-peaked phytoliths can be used to distinguish domesticated rice from wild rice based on multivariate linear discriminant function analysis and three-dimensional measurements [19, 20]. However, very few double-peaked phytoliths have been found in prehistoric rice soil or sediments [21, 22].

Rice bulliform phytoliths are abundant and unique to the rice leaf, with the presence of fish-scale decorations on fan edges (bulliform phytoliths) [18]. Bulliform phytolith measurement is a method that could potentially be used to distinguish domesticated from wild rice. However, previous studies on rice plants and their relatives have suggested that bulliform measurement alone is unable to distinguish wild *Oryza* species from domesticated ones [19, 23–25].

Another method for discriminating bulliform phytoliths of wild and domesticated rice is based on the number of scales on fan edges. Fujiwara [26] was the first to find that fish-scale decorations are different in wild and domesticated rice. The scales of domesticated rice bulliform phytoliths are larger and have irregular shapes, while those of wild rice bulliform phytoliths look like a tortoise shell. However, this kind of qualitative discrimination is hard to apply to research in practice and it is thus important to explore quantitative standards.

Lu et al. [22] studied the number of fish-scale decorations on the rim of rice bulliform phytoliths from seven species of wild rice and six species of domesticated rice, finding that bulliform phytoliths of domesticated rice species generally had 8–14 fish-scale decorations, while those belonging to varieties of wild rice commonly had <9. It would thus seem that bulliform phytoliths with ≥9 decorations would be a useful standard for identifying domesticated species.

In practice, however, because of the overlap in the number of scale decorations between wild and domesticated rice species, a single bulliform phytolith is not sufficient to enable a distinction to be made between domesticated and wild rice species. Moreover, in locations where wild and domesticated forms of rice are likely to have overlapped, determining the precise source of fossil phytoliths recovered from sedimentary records can be problematic [23]. For these reasons, more clear specification is needed.

This study on domesticated and wild rice paddy surface soil suggests that the proportion of bulliform phytoliths with ≥9 decorations can be adopted as a criterion to discriminate between wild and domesticated rice.

## Materials and Methods

**Fig 1:**
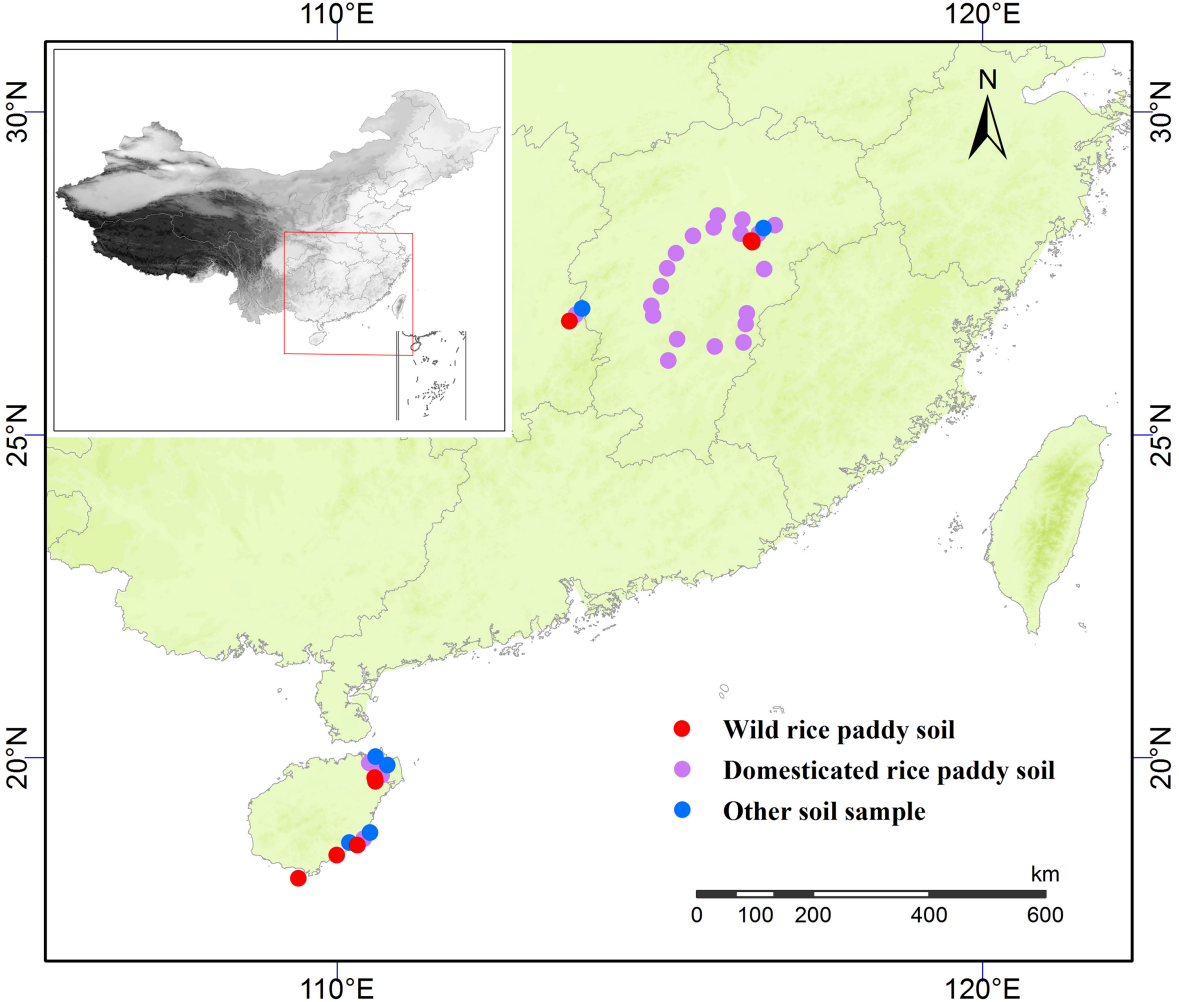
Geographic locations of sample collection sites. (map modified from Grass GIS; https://grass.osgeo.org/); red dots represent wild rice paddy soil samples, purple dots represent domesticated rice paddy soil samples, and blue dots represent other soil samples.

Seventy-one samples of surface rice soil from south China, including from Hainan, Hu’nan, and Jiangxi Provinces, were collected for phytolith analysis (Fig 1). These included 29 surface soil samples from 9 wild rice fields, 30 surface soil samples from 24 modern rice paddies, and 12 soil samples from 9 other fields. Details of all samples are given in Table 1. All necessary permits for the described samples from Jiangxi Province were obtained from the Jiangxi Academy of Agricultural Sciences. No permits for other samples were needed.

**Table 1:** List of samples used in the study.

No.	Code	Source	Location	Longitude (°E)	Latitude (°N)
1	DY1	Wild rice field	Dongxiang County, Jiangxi Province	116.533	28.083
2	DY2	Wild rice field	Dongxiang County, Jiangxi Province	116.533	28.1
3	DY3	Wild rice field	Dongxiang County, Jiangxi Province	116.533	28.083
4	HL-BT1	Wild rice field	Wenchang City, Hainan Province	110.681	19.787
5	HL-BT2	Wild rice field	Wenchang City, Hainan Province	110.681	19.787
6	TS-BT4	Wild rice field	Wenchang City, Hainan Province	110.694	19.727
7	WN-2	Wild rice field	Wanning City, Hainan Province	110.411	18.741
8	WN-4	Wild rice field	Wanning City, Hainan Province	110.411	18.741
9	WN-5	Wild rice field	Wanning City, Hainan Province	110.411	18.741
10	WN-BT6	Wild rice field	Wanning City, Hainan Province	110.411	18.741
11	WN-BT7	Wild rice field	Wanning City, Hainan Province	110.411	18.741
12	WN-BT8	Wild rice field	Wanning City, Hainan Province	110.411	18.741
13	WN-BT9	Wild rice field	Wanning City, Hainan Province	110.411	18.741
14	DXA	Wild rice field	Dongxiang County, Jiangxi Province	116.528	28.108
15	DXS-1	Wild rice field	Dongxiang County, Jiangxi Province	116.509	28.104
16	DXS-2	Wild rice field	Dongxiang County, Jiangxi Province	116.509	28.104
17	10CL-B1	Wild rice field	Chaling County, Hunan Province	113.696	26.861
18	10CL-B3	Wild rice field	Chaling County, Hunan Province	113.696	26.861
19	10CL-B4	Wild rice field	Chaling County, Hunan Province	113.696	26.861
20	10CL-B5	Wild rice field	Chaling County, Hunan Province	113.697	26.861
21	10CL-B6	Wild rice field	Chaling County, Hunan Province	113.697	26.861
22	10CL-B7	Wild rice field	Chaling County, Hunan Province	113.696	26.862
23	10CL-B8	Wild rice field	Chaling County, Hunan Province	113.696	26.861
24	10CL-B9	Wild rice field	Chaling County, Hunan Province	113.697	26.861
25	10CL-B10	Wild rice field	Chaling County, Hunan Province	113.697	26.861
26	10CL-S4	Wild rice field	Chaling County, Hunan Province	113.696	26.861
27	ZX-2	*Oryza officinalis* field topsoil	Lingshui County, Hainan Province	110.095	18.589
28	ZX-3	Edge of *Oryza officinalis* field	Lingshui County, Hainan Province	110.095	18.589
29	LHT-2	*Oryza* granulate field topsoil	Sanya City, Hainan Province	109.499	18.226
30	JX01	Domesticated rice paddy	Dongxiang County, Jiangxi Province	116.533	28.117
31	JX02	Domesticated rice paddy	Yujiang County, Jiangxi Province	116.783	28.25
32	JX03	Domesticated rice paddy	Jinxian County, Jiangxi Province	116.283	28.333
33	JX04	Domesticated rice paddy	Gan County, Jiangxi Province	115.133	26.15
34	JX05	Domesticated rice paddy	Gan County, Jiangxi Province	115.133	26.15
35	JX06	Domesticated rice paddy	Nanchang County, Jiangxi Province	115.9	28.4
36	JX07	Domesticated rice paddy	Fengcheng City, Jiangxi Province	115.833	28.217
37	JX08	Domesticated rice paddy	Zhangshu City, Jiangxi Province	115.517	28.083
38	JX09	Domesticated rice paddy	Xingan County, Jiangxi Province	115.25	27.817
39	JX10	Domesticated rice paddy	Xiajiang County, Jiangxi Province	115.117	27.583
40	JX11	Domesticated rice paddy	Jishui County, Jiangxi Province	115.017	27.301
41	JX12	Domesticated rice paddy	Ji’an County, Jiangxi Province	114.867	27
42	JX13	Domesticated rice paddy	Taihe County, Jiangxi Province	114.9	26.85
43	JX14	Domesticated rice paddy	Xingguo County, Jiangxi Province	115.267	26.483
44	JX15	Domesticated rice paddy	Ningdu County, Jiangxi Province	115.85	26.367
45	JX16	Domesticated rice paddy	Shicheng County, Jiangxi Province	116.3	26.433
46	JX17	Domesticated rice paddy	Guangchang County, Jiangxi Province	116.333	26.717
47	JX18	Domesticated rice paddy	Guangchang County, Jiangxi Province	116.35	26.883
48	JX19	Domesticated rice paddy	Nancheng County, Jiangxi Province	116.617	27.567
49	JX20	Domesticated rice paddy	Fuzhou County, Jiangxi Province	116.25	28.117
50	HKML-1	Domesticated rice paddy	Haikou City, Hainan Province	110.498	19.918
51	HL-OS1	Domesticated rice paddy	Wenchang City, Hainan Province	110.681	19.787
52	TS-BT1	Domesticated rice paddy	Wenchang City, Hainan Province	110.694	19.727
53	TS-BT2	Domesticated rice paddy	Wenchang City, Hainan Province	110.694	19.727
54	TS-BT3	Domesticated rice paddy	Wenchang City, Hainan Province	110.694	19.727
55	WN-BT1	Domesticated rice paddy	Wanning City, Hainan Province	110.411	18.741
56	WN-BT2	Domesticated rice paddy	Wanning City, Hainan Province	110.411	18.741
57	WN-BT3	Domesticated rice paddy	Wanning City, Hainan Province	110.411	18.741
58	WN-BT4	Domesticated rice paddy	Wanning City, Hainan Province	110.411	18.741
59	10CL	Domesticated rice paddy	Chaling County, Hunan Province	113.696	26.861
60	ZX-4	Natural topsoil	Lingshui County, Hainan Province	110.095	18.589
61	HKML-2	Bushwood topsoil	Haikou City, Hainan Province	110.498	19.918
62	10CL	Broad-leaved forest topsoil	Chaling County, Hunan Province	113.696	26.861
63	HL-OS2	Edge of wild rice paddy	Wenchang City, Hainan Province	110.681	19.787
64	WN-BT5	Edge of wild rice paddy	Wanning City, Hainan Province	110.411	18.741
65	WN-BT10	Edge of wild rice paddy	Wanning City, Hainan Province	110.411	18.741
66	DXS	Edge of wild rice paddy	Dongxiang County, Jiangxi Province	116.509	28.104
67	10CL-S1	Soil around wild rice root	Chaling County, Hunan Province	113.696	26.861
68	10CL-S2	Soil around wild rice root	Chaling County, Hunan Province	113.696	26.861
69	10CL-S3	Soil around wild rice root	Chaling County, Hunan Province	113.696	26.861
70	10CL-FB1	Natural topsoil	Chaling County, Hunan Province	113.696	26.861
71	10CL-FB2	No wild rice area	Chaling County, Hunan Province	113.696	26.861

Extraction of phytoliths from soil samples followed procedures described by Zhang [27], with slight modifications. Initially, 5 g of soil were weighed. Subsequently, 30% hydrogen peroxide (H_2_O_2_) and cold 15% hydrochloric acid (HCl) were added to each sample to remove organic matter and carbonates. The samples were then subjected to heavy liquid flotation using zinc bromide (ZnBr_2_, density 2.35 g/cm^3^) to separate phytoliths, with these mounted on a slide with Canada Balsam.

**Fig 2:**
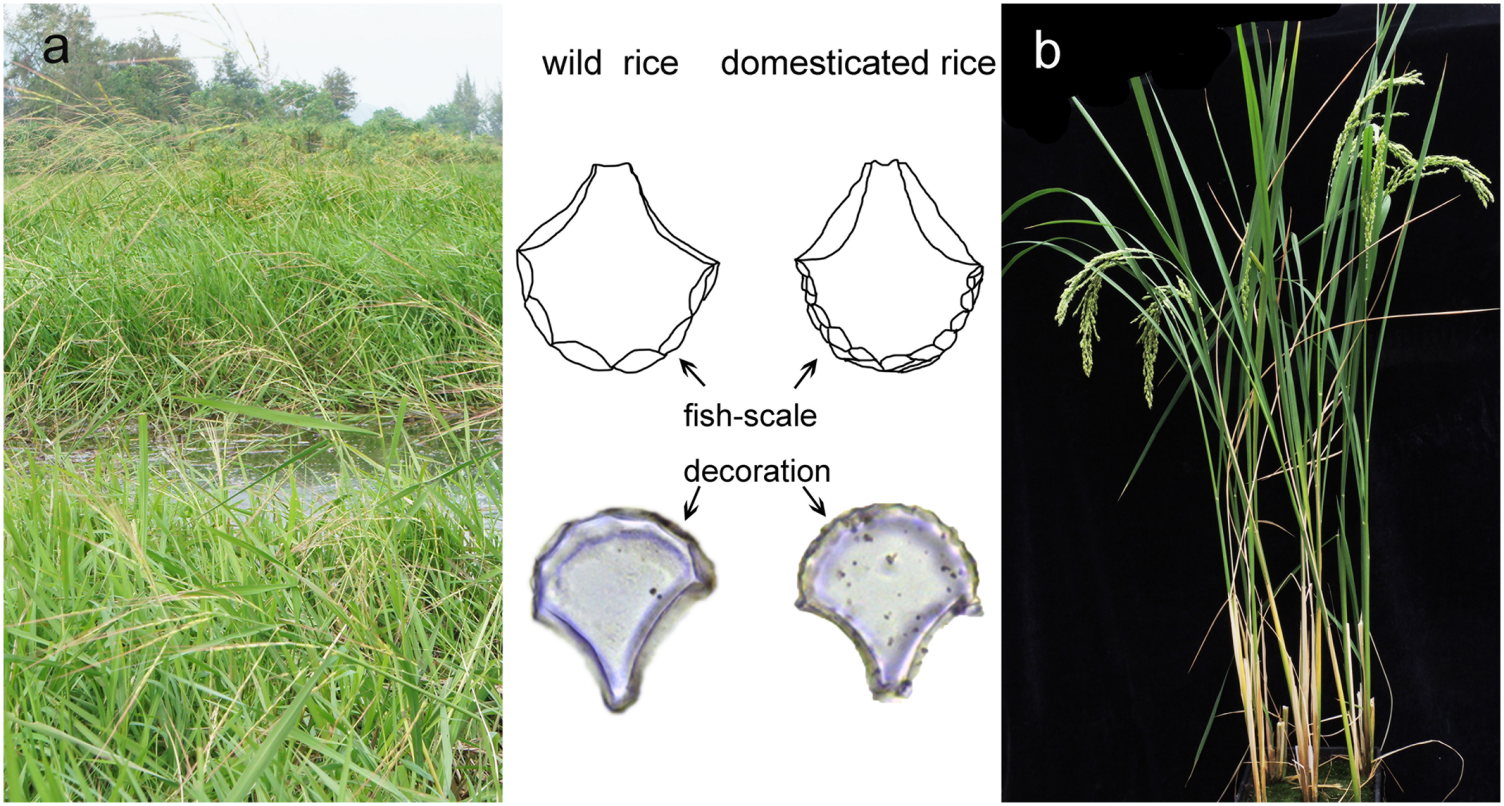
Fish-scale decorations in rice bulliform phytoliths (Modified as per [22] [30]). a: wild rice plant and its growing environment; b: domesticated rice plant.

Phytolith counting and identification were performed using a Leica microscope with phase-contrast at 400X magnification. The bulliform phytolith selection criteria were modified from Wang & Lu [28] (both symmetric and asymmetric ones). Phytoliths <10 um in size were excluded because of the inability to clearly count decorations. For each rice bulliform phytolith, the number of fish-scale decorations around the edge of fan-shaped phytoliths (Fig 2) was counted. Each sample was scanned until 50 rice bulliform phytoliths were encountered [29]. In each case, the proportion of rice bulliform phytoliths with ≥9 fish-scale decorations was calculated.

## Results

**Fig 3:**
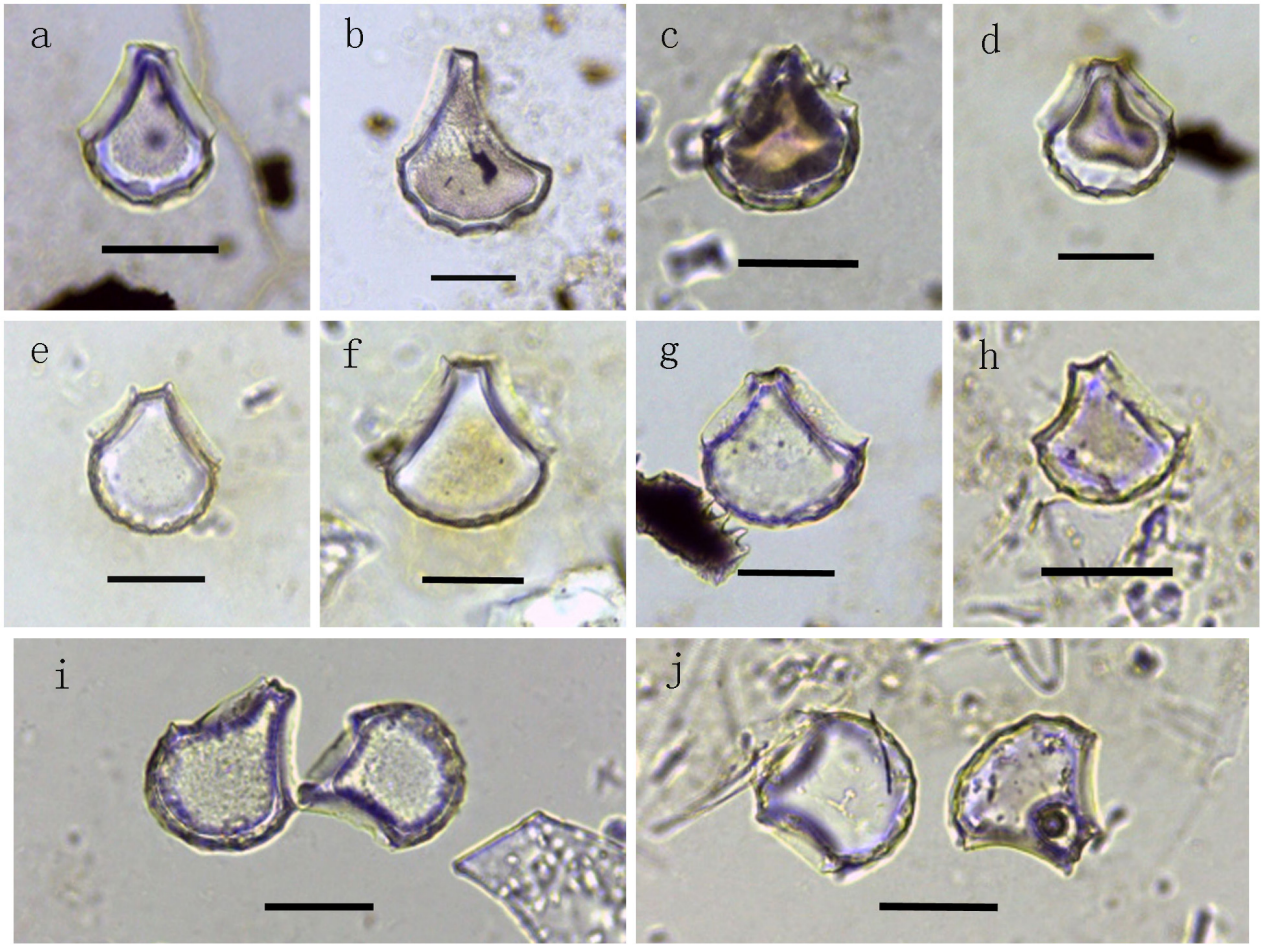
Bulliform phytoliths in sample rice (10 um). a-c: bulliform phytoliths from wild rice fields with <9 fish-scale decorations; d: bulliform phytoliths from wild rice field with ≥9 fish-scale decorations; e-g: bulliform phytoliths from modern rice paddies with ≥9 fish-scale decorations; h: bulliform phytolith from modern rice paddies with <9 fish-scale decorations; i: bulliform phytolith from wild rice field; j: bulliform phytolith from modern rice paddy.

Bulliform phytoliths in soil samples were mostly well preserved, enabling correct identification (Fig 3 and S1 Table). Abundant rice phytoliths (including bulliform phytoliths and bilobate phytoliths) were found, but these included almost no double-peaked phytoliths.

**Fig 4:**
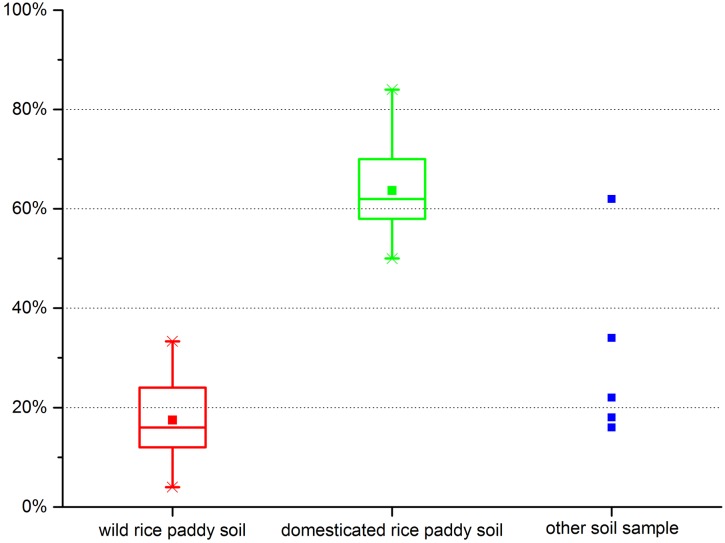
The proportion of bulliform phytoliths with ≥9 fish-scale decorations in samples.

*Bulliform phytoliths from wild rice field—*Of all 29 wild rice soil samples, no rice bulliform phytoliths were found in samples Nos. 20, 25, and 29. In the other 26 wild rice soil samples, the highest proportion of bulliform phytoliths with ≥9 fish-scale decorations was 33.33% (No. 9), while the lowest proportion was 4% (Nos. 1 and 24); the average proportion in the 26 wild rice soil samples was 17.46% ± 8.29% (Fig 4, left).

*Bulliform phytoliths from domesticated rice paddies—*Rice bulliform phytoliths were found in all 30 domesticated rice paddy soil samples. The highest proportion of bulliform phytoliths with ≥9 fish-scale decorations was 84% (No. 59), while the lowest proportion was 50% (No. 42); the average proportion in all 30 soil samples from domesticated rice paddies was 63.70% ± 9.22% (Fig 4, middle).

*Bulliform phytoliths from other fields—*Rice bulliform phytoliths were found in only five other field soil samples (Fig 4, right). Of these, the highest proportion of bulliform phytoliths with ≥9 fish-scale decorations was 62% (No. 63), while the lowest was 16% (No. 71). Although the five soil samples were neither from wild rice fields nor from domesticated rice paddies, their locations were very close to paddy or wild rice fields, so it is possible that these samples contained rice bulliform phytoliths because of soil tilling or other disturbance activities.

**Fig 5:**
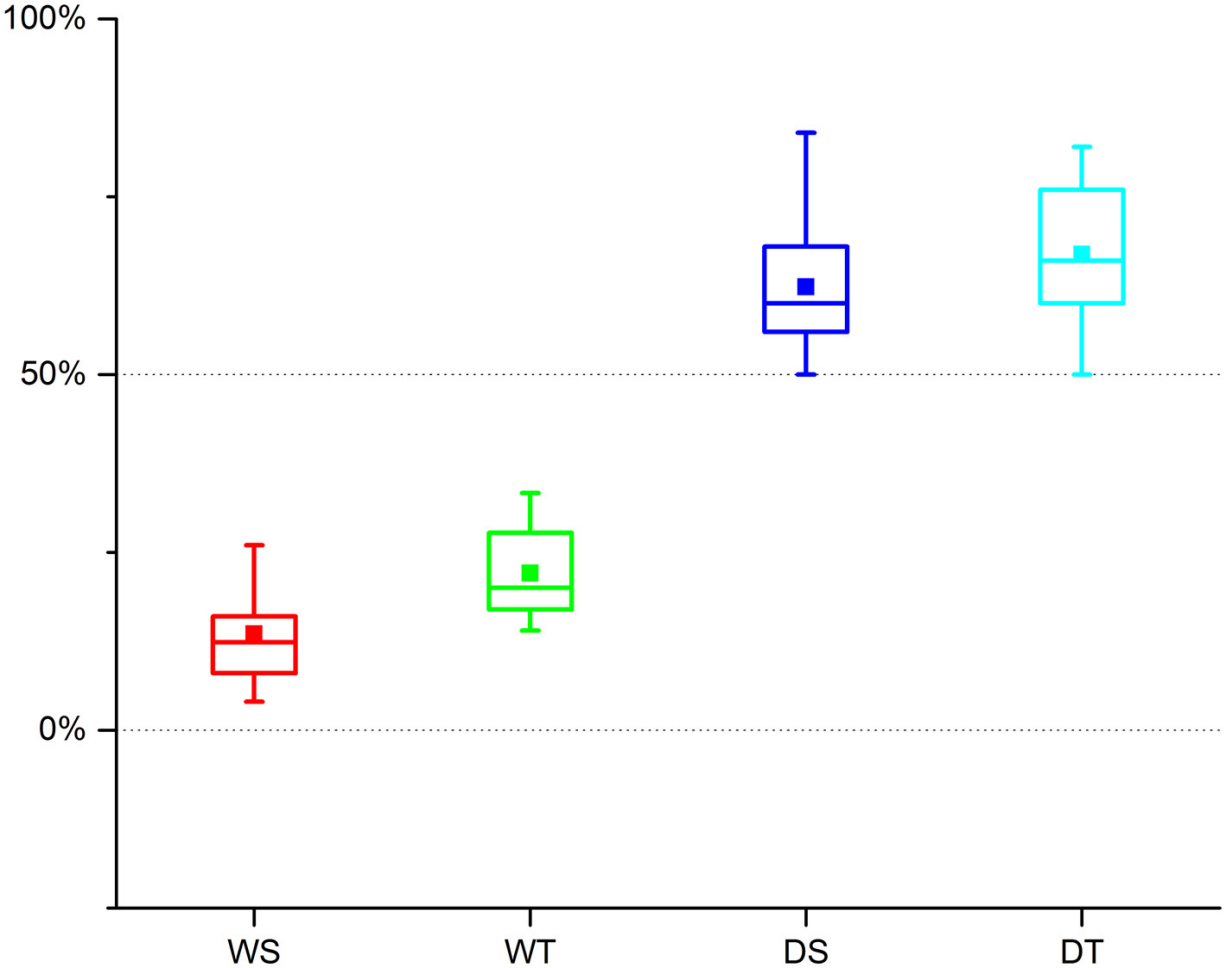
The proportion of bulliform phytoliths with ≥9 fish-scale decorations from different climatic regions. WS: wild rice field soil in subtropical region; WT: wild rice field soil in tropical region. DS: domesticated rice paddy soil in subtropical region; DT: domesticated rice paddy soil in tropical region.

We also analyzed the proportion of bulliform phytoliths with ≥9 fish-scale decorations from different climatic regions. Jiangxi and Hunan Provinces are subtropical, while Hainan Province is located in the tropics. Table 2 shows the number of samples from different climatic regions. From Fig 5, we can see that in wild rice soil samples, the highest proportions of bulliform phytoliths with ≥9 fish-scale decorations were found in samples from the tropics, and the lowest proportions of bulliform phytoliths with ≥9 fish-scale decorations were found in samples from the subtropical region. Moreover, as shown from the analysis of the box height in Fig 5, the proportions from both wild and domesticated paddy soil samples from the tropics were more scattered than in the case of samples from the subtropical region.

**Table 2:** Number of samples from different climatic regions.

	subtropical region	tropical region
wild rice field soil sample	16	13
domesticated rice paddy soil sample	21	9

## Discussion

From our study, it is evident that the indicator—proportion of bulliform phytoliths with ≥9 fish-scale decorations—can be used to clearly discriminate wild and domesticated rice. We therefore believe that when the proportion of bulliform phytoliths with ≥9 fish-scale decorations is higher than 63.70% ± 9.22%, the sample can be regarded as domesticated rice; when the proportion of bulliform phytoliths with ≥9 fish-scale decorations is less than 17.46% ± 8.29%, the sample can be classified as wild rice.

Previous studies have often employed morphometric parameters (length, width and b/a—ratio of length of handle to fan) of bulliform phytoliths to analyze ancient rice remains [31–38]. Some researchers have established discriminant equations to differentiate between *japonica* and *indica* rice [39–41]. However, Wang et al. verified these discriminant equations and found that wild rice was always misjudged; for this reason, when using discriminant equations to determine the origin of archaeological samples, extreme caution is required [28]. Moreover, Gu et al. found that three-dimensional morphological features of bulliform phytoliths from *Oryza sativa* are scattered, with significant overlap of this species with its relatives. Due to this wide overlap, bulliform phytolith measurement alone cannot be used to distinguish wild *Oryza* species from domesticated ones [19].

The fish-scale decoration features in single bulliform phytolith have shown great potential in previous studies [22, 27, 42–45]. However, use of these features in single bulliform phytoliths to distinguish wild/domesticated rice remains controversial. One important reason is that the number of fish-scale decorations overlaps across species, so that it is not possible to use a single bulliform phytolith to classify rice properties.

In order to improve the validity of this method, in this study we examined at least 50 phytoliths from each sample to calculate the proportion of bulliform phytoliths with ≥9 fish-scale decorations. This method can help avoid the uncertainty inherent in single phytolith analysis. Besides, bulliform phytoliths are abundant in the genus *Oryza* and previous research has shown that the highest silica percentage is present in the leaf blade [46]. It is therefore feasible to discriminate wild and domesticated rice through the number of fish-scale decorations found around the bulliform phytolith.

Bulliform cells (motor cells), situated in the upper epidermis of leaf blades [47], are water storage mechanisms and play a role in mature leaf rolling and/or folding in case of water stress [48, 49]. During periods of excessive water loss, the bulliform cells became flaccid and enable the leaf to roll in order to maintain water; under sufficient water conditions, bulliform cells are filled with water and expand, and the blade thus flattens [50]. Leaf rolling can affect light interception of the base and enhance the ability to resist water stress [50, 51].

Bulliform cells are closely associated with adjacent colorless cells [52]. Their morphology, combined with that of enlarged colorless cells, has been used as a taxonomic characteristic [53]. The colorless cells are smaller than the bulliform cells, translucent, voluminous, highly vacuolized, and arranged in uniseriate columns connecting the abaxial epidermis and bulliform cells [52]. The colorless cells are variable in shape and size [53]. Fish-scale decorations on the bulliform phytolith are comprised of cavities squeezed by colorless cells [28] and leaf curling will increase the number of cavities. Wild rice usually grows in swampy conditions [54], where water is abundant (Fig 2, left), and so leaves curl less; on the contrary, domesticated rice leaves are erect and distant from water (Fig 2, right), and so the leaves need to curl repeatedly to hold water. This might explain why bulliform phytoliths of domesticated rice have more fish-scale decorations than those of wild rice.

In our study, the proportion of bulliform phytoliths with ≥9 fish-scale decorations in samples from different climatic regions was analyzed. We found that in subtropical and tropical regions, the proportion of bulliform phytoliths with ≥9 fish-scale decorations in wild rice field soil samples was notably different, while the difference was smaller in the case of domesticated rice paddy samples; it is unclear whether the difference in the former case is climate-related. All domesticated rice paddy samples collected in the study came from southern China; however, since northern China is also a main rice growing area, we hope to study more soil samples from northern China in future.

Admittedly, the discrimination of wild and domesticated bulliform phytoliths might be influenced by other factors, such as erosion and dissolution of bulliform phytoliths [55], making it more difficult to precisely count the number of fish-scale decorations. In addition, hybridization of wild rice and domesticated rice species would also affect the ability to discriminate between wild/domesticated rice. We, therefore, recommend further research on wild-domesticated hybridization, which should help understand the variation in decorations and document cultivation and domestication.

## Conclusion

This study systematically analyzed differences in bulliform phytolith fish-scale decoration numbers between domesticated rice paddy soil and wild rice field soil in South China. Results showed that, in domesticated rice soil, the proportion of bulliform phytoliths with ≥9 fish-scale decorations was higher than 63.70% ± 9.22%, while in wild rice soil, the proportion was less than 17.46% ± 8.29%. The study therefore indicates that the proportion of bulliform phytoliths with ≥9 fish-scale decorations can be used to successfully discriminate between wild and domesticated rice. This provides significant insights for research into rice origin and domestication.

## Supporting Information

S1 Table The proportion of bulliform phytoliths with ≥9 fish-scale decorations. (XLSX)
